# Mesenchymal Stem Cells Exhibit Firm Adhesion, Crawling, Spreading and Transmigration across Aortic Endothelial Cells: Effects of Chemokines and Shear

**DOI:** 10.1371/journal.pone.0025663

**Published:** 2011-09-28

**Authors:** Giselle Chamberlain, Helen Smith, G. Ed Rainger, Jim Middleton

**Affiliations:** 1 Leopold Muller Arthritis Research Centre, Medical School, Keele University, RJAH Orthopaedic Hospital, Oswestry, Shropshire, United Kingdom; 2 School of Clinical and Experimental Medicine, College of Medicine and Dentistry, University of Birmingham, Birmingham, United Kingdom; 3 Faculty of Medicine and Dentistry, School of Oral and Dental Sciences, University of Bristol, Bristol, United Kingdom; Centro Cardiologico Monzino, Italy

## Abstract

Mesenchymal stem cells (MSCs) have anti-inflammatory and immunosuppressive properties and may be useful in the therapy of diseases such as arteriosclerosis. MSCs have some ability to traffic into inflamed tissues, however to exploit this therapeutically their migratory mechanisms need to be elucidated. This study examines the interaction of murine MSCs (mMSCs) with, and their migration across, murine aortic endothelial cells (MAECs), and the effects of chemokines and shear stress. The interaction of mMSCs with MAECs was examined under physiological flow conditions. mMSCs showed lack of interaction with MAECs under continuous flow. However, when the flow was stopped (for 10min) and then started, mMSCs adhered and crawled on the endothelial surface, extending fine microvillous processes (filopodia). They then spread extending pseudopodia in multiple directions. CXCL9 significantly enhanced the percentage of mMSCs adhering, crawling and spreading and shear forces markedly stimulated crawling and spreading. CXCL9, CXCL16, CCL20 and CCL25 significantly enhanced transendothelial migration across MAECs. The transmigrated mMSCs had down-regulated receptors CXCR3, CXCR6, CCR6 and CCR9. This study furthers the knowledge of MSC transendothelial migration and the effects of chemokines and shear stress which is of relevance to inflammatory diseases such as arteriosclerosis.

## Introduction

The ability of mesenchymal stem cells (MSCs) to differentiate down several different cell lineages, as well as their anti-inflammatory and immunologic characteristics, their lack of ethical controversy, and their relative ease of expansion in culture make these cells a promising source of stem cells for treatment of many forms of inflammatory disease and injury [1]. This makes them potentially useful as an anti-inflammatory therapy for atherosclerosis and several reports have used cultured MSCs to treat myocardial infarction [2]–[4], as well as other conditions such as stroke or spinal cord injury [5], [6], experimental autoimmune encephalitis (EAE) [7], radiation injury [8], wounded skin [9] and graft-versus-host disease [10]. Although site-directed delivery of MSCs may be useful in certain settings, for example to treat non-union fractures [11], systemic infusion of MSCs circumvents problems associated with site-specific delivery, such as calcification and tissue damage [12]. Furthermore, systemic delivery enables the delivery of multiple doses, and is potentially a less invasive procedure than site-directed delivery. In order to treat atherosclerosis directly via systemic delivery, MSCs would need to traverse the aortic endothelium and enter the tissue to exert anti-inflammatory effects. However, the migration and engraftment of MSCs by this route is not very efficient [13], and there is a need to understand how MSCs extravasate from the blood into tissues so that this recruitment may be increased to help reduce inflammation and enhance tissue repair.

Much is known concerning the leukocyte adhesion cascade and how these cells migrate from the circulation and into inflamed tissues [14], [15]. Leukocytes undergo a sequence of interactions at the luminal endothelial surface including tethering, rolling, activation, arrest, spreading and crawling, followed by transendothelial migration. However, there is a lack of knowledge concerning MSCs, whether they undergo similar interactions with endothelial cells and what factors regulate their transendothelial migration. Furthermore the influence fluid shear stress, which occurs physiologically in the circulation, on MSC-endothelial interactions needs to be addressed as this has been shown to be important for leukocyte migration [16].

Chemokine receptors and their chemokine ligands are essential components involved in the migration of leukocytes into sites of inflammation, and we have recently demonstrated functional expression of various chemokine receptors on murine MSCs (mMSCs) using standard Boyden-type chambers in the absence of endothelial cells [17]. The expression of chemokine receptors on human MSCs have also been reported by ourselves and others [17]–[22], some of which are in common with the mouse (CXCR3, CXCR6 and CCR9). There are also numerous leukocyte adhesion molecules known to be involved in migration of cells across the endothelium, some of which are also reported to be expressed on MSCs [23], [24]. Adhesion molecule pairs that may be functionally important in the adherence of MSCs to the endothelium are CD44, VCAM-1 and its counterligand VLA-4, and other β1 integrins [25]–[28]. However, little is known about the mechanism of MSC transendothelial migration and the role of chemokines in driving this mechanism. Two recent studies have examined transendothelial migration under static conditions *in vitro* using a co-culture of endothelial cells and MSCs [27], [29]. They both found that when endothelial cells were stimulated with inflammatory cytokines, MSCs showed morphological changes and integration within the endothelial monolayer. They also found that MSCs penetrate the endothelium via plasmic podia, and secrete MMP-2, a basement membrane-degrading enzyme that is known to facilitate the trafficking of haematopoietic stem cells [30].

The aim of the present study was to investigate the effect of chemokines and shear stress on the adherence murine MSCs (mMSCs) to murine aortic endothelial cells (MAECs). It is shown for the first time that MSCs undergo spreading and crawling behaviour when in contact with endothelial cells and that these are enhanced by chemokine stimulation and shear stress. Chemokines also enhance the transendothelial migration of mMSCs across MAECs which results in down-regulation of chemokine receptors on migrated cells. We have previously shown that mMSCs express some of the same chemokine receptors as human MSCs [17] and consequently these mMSCs would be a useful model to further study the role of selective chemokine receptors in *in vivo* models of atherosclerosis and myocardial infarction.

## Methods

### Isolation and Expansion of Murine MSCs

Primary mMSCs were obtained from BALB/c mice, 6–10 weeks old [17] and isolated as previously described [17], [31], with ethical approval sought from the ethics committee of the RJAH Orthopaedic Hospital, Oswestry, UK. Briefly, marrow was removed from the long bones and cells plated out in cell isolation media (CIM) (RPMI-1640 (Lonza, Slough, UK) with 9% FBS, 9% horse serum (both Gibco, Invitrogen, Paisley, UK) at 37°C, 5% CO_2_. After 24 hours, non-adherent cells were removed. After 4 weeks cells were re-plated at 100 cells per cm^2^ in complete expansion media (CEM) (Iscove Modified Dulbecco Medium (Lonza) with 9% FBS, 9% horse serum) to expand MSCs. These cells were >95% positive for CD105, and completely negative (0%) for CD45 and CD34 [17]. When incubated in osteogenic and adipogenic media they were positive for alkaline phosphatase and intracellular lipid respectively [17].

### Culture of Mouse Endothelial Cells

The MAEC line was a kind gift from Dr Hiroko Inoue, Tsurumi University School of Dental Medicine, Japan. The cell line was established from p53-deficient mouse aortas [32]. It has an endothelial phenotype, with Weibel-Palade bodies and endothelial markers and adhesion molecules. Furthermore TNFα promotes lymphocyte adhesion to MAECs, providing a model to study inflammation and leukocyte migration *in vitro.* Cells were grown in Medium 199 (Sigma, Poole, UK) with 5% FBS, 1 U/ml of heparin sodium and 5 ng/ml of murine VEGF (Peprotech, London, UK), at 37°C, 5% CO_2_. Media was changed every 3–4 days.

The brain endothelial cell line was purchased from LGC Promochem (ATCC)(Teddington, UK) and was originally isolated from a BALB/c mouse brain endothelioma [33], [34]. The endothelial nature of the cells was confirmed by the observed expression of von Willebrand factor and uptake of fluorescently labeled LDL. Other molecules expressed by endothelial cells have been shown to be constitutively expressed by these cells, including ICAM-1 and VCAM-1, or induced by stimulation with TNFα or lipopolysaccharide (LPS), such as E-selectin and P-selectin. Cells were grown in DMEM (Lonza) with added antibiotics and 10% FBS according to the manufacturer's instructions.

### Flow Assay

MAECs were seeded into Ibidi microslides (Wolf laboratories, Pocklington, UK) and grown to confluence, then stimulated with murine TNFα at 100 ng/ml for 16 hours at 37°C. They were then washed with medium and treated with or without murine CXCL9 (MIG) (100 ng/ml for 30 min)(Peprotech). The microslides were incorporated into a flow-based adhesion assay where a syringe pump draws liquid through the microslides at a controlled rate, at 37°C, whilst allowing visualisation of the cells inside the slide by phase contrast microscopy [35]. Murine MSCs in serum-free medium were perfused (1×10^6^/ml) through the microslides at 0.1 Pa for 4 minutes, then washed with medium at the same flow rate for 15 minutes. Video recordings were made throughout to analyse MSC behaviour. Murine MSCs were also added to the microslides at 4×10^6^/ml and allowed to incubate at 37°C for 10 minutes, under static conditions, then serum-free medium was perfused through at 0.1 Pa (1 dyne/cm^2^) for 2 hours. Images were recorded every 15 seconds throughout to examine interaction of MSCs with the endothelium.

### Transendothelial Chemotaxis

Transwell inserts with 8 µm pore size filters were coated with fibronectin (from human plasma, Sigma) (4 ug/ml in PBS) and transferred to 24-well plates treated with Sigmacote (Sigma), to prevent the adherence of migrated cells. Approximately 4×10^5^ MAECs were seeded onto the filter of each transwell insert and grown to 100% confluence over 2 days, then activated with 100 ng/ml of murine TNFα (Peprotech) in serum-free medium for 4 hours at 37°C. Three wells were left unstimulated as a control. Murine MSCs were stained with 8 µm Calcein-AM (Molecular Probes, Invitrogen) in PBS for 30 min at 37°C. Meanwhile, medium with or without murine CXCL9 (MIG), CCL20 (MIP3α), CCL25 (TECK) and CXCL16 (Peprotech) were added to the wells of a 24-well plate, underneath the inserts, at various concentrations. Each condition was performed in triplicate. 600,000 mMSCs were added to each of the upper wells of the inserts, suspended in serum-free medium, and the plate incubated at 37°C, 5% CO_2_ and 90% humidity overnight for 16 hours. The number of mMSCs which had migrated through to the bottom well was calculated using an FLX800 microplate fluorescence reader (Bio-tek Instruments Ltd, Potton, UK), having set up a standard curve for fluorescence versus cell number.

The amount of each chemokine used was 10 ng/ml. This was based on initial dose response experiments (0–1000 ng/ml) using the same murine MSCs in Boyden-type chemotaxis assays [17]. Results showed that 10 ng/ml or 100 ng/ml were the most effective concentrations to significantly stimulate MSC migration; lower (<10 ng/ml) or higher (>500 ng/ml) concentrations of each chemokine did not stimulate migration [17]. Therefore in the current transendothelial migration experiments 10 and 100 ng/ml of chemokines were used and the former concentration was found to be effective at stimulating MSC migration whereas the latter produced insignificant results.

### Flow Cytometry

MSCs from transendothelial chemotaxis experiments or those detached from flasks by incubation with trypsin-EDTA were resuspended in buffer (PBS + 2% BSA) containing 10% of an appropriate serum to block non-specific binding for 60 minutes at 4°C. Cells were found to be >95% viable using the trypan blue exclusion assay. MSCs were incubated on ice for 30 minutes with saturating amounts of the appropriate primary antibody in buffer, washed, then stained with an appropriate biotinylated secondary antibody for 30 minutes on ice, washed, then stained with a streptavidin-PE labelled conjugate for 30 minutes [17]. As a negative control, cells were also stained with the relevant isotype control immunoglobulin (Ig) instead of primary antibody, as well as the secondary antibody and streptavidin conjugate described. Antibodies were anti-mouse CCR6, anti-mouse CCR9, anti-mouse CXCR3, anti-mouse CXCR6, rat IgG2a isotype control and rat IgG2b isotype control (all R&D Systems, Abingdon, UK), and biotinylated anti-rat Ig and streptavidin-PE conjugate (both BD Pharmingen, Oxford, UK). For the transendothelial chemotaxis experiments, the percentage of MSCs positive for each of the chemokine receptors was determined by gating only on calcein labelled cells in the FITC/FL1 channel, and this gating was also used for the mean fluorescence intensity (MFI) measurements after subtraction of the Ig control.

### Statistics

For transendothelial migration of mMSCs in response to chemokines ANOVA was performed followed by pair wise comparisons with control using the Dunnett's test. For analysis of video data of the percentage of mMSCs adhered, crawled and spread, unpaired T tests were used to compare chemokine treatments with control.

## Results

### Murine Mesenchymal Stem Cell Interaction with TNFα-Stimulated Endothelium under Shear Flow Conditions

Microslides were seeded with MAECs and stimulated with TNFα (100 ng/ml) as described. MSCs were flowed through the microslides at 0.1 Pa, which is a physiological flow rate relevant to leukocyte recruitment in the postcapillary venules or the low shear environments in the arterial circulation which are prone to formation of atheroma. MSCs were observed for any interaction with the endothelium. Over 4 minutes of mMSCs flowing through the microslides there was no interaction of MSCs with the endothelium. No mMSCs were observed to be rolling on or adhered to the endothelial layer both after treating these cells with and without TNFα. This assay was repeated with MAECs with and without TNFα stimulation, together with or without the addition of the chemokines CXCL9, CXCL16, CCL20 and CCL25 at 100 ng/ml for 30 minutes; these chemokines were chosen since they are ligands for the chemokine receptors previously shown to be present on mMSCs [17]. Again no mMSCs were seen to interact with the endothelium after stimulation with any combination of TNFα and/or chemokines (Video S1). The flow rate was slowed down to less than half the speed, i.e. 0.15 ml/min (0.05 Pa, 0.5 dynes/cm^2^), but mMSCs still did not roll on or adhere to the endothelium in the presence or absence of chemokines. Similarly when murine brain endothelial cells, bEnd.3 cells, were used instead of MAECs there was no rolling or adherence of mMSCs to these cells under flow conditions (data not shown).

**Figure 1:**
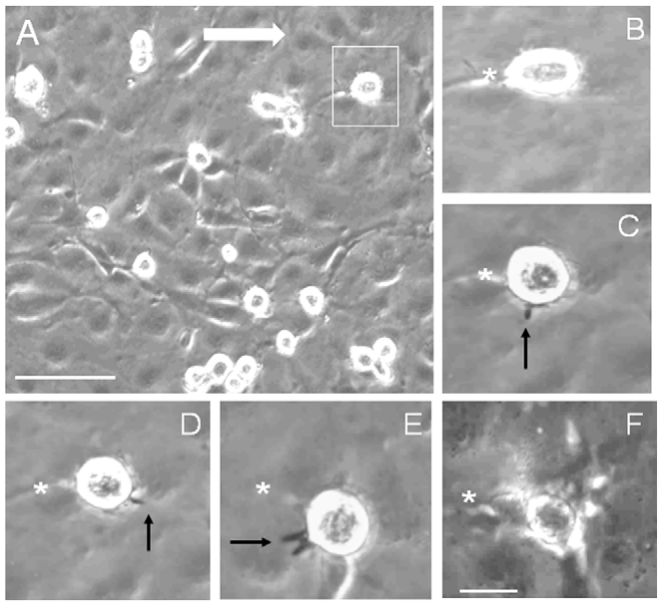
Still images from flow assay video to illustrate crawling of mMSCs on mouse aortic endothelial cells (MAECs). MAECs were TNFα-activated, mMSCs added and left for 10 minutes. Flow was then started and continued for 2 hours and video recordings made. The direction of flow is shown by the large white arrow. A, shows a low power image of TNFα-activated MAECs with adherent phase bright MSCs immediately after commencing shear stress (from video S2). B-F show detail of an MSC indicated in the top right of A. B is immediately after commencing shear stress and C, D, E and F after 30, 60, 80 and 120 minutes of flow respectively. The asterisk is a reference point to illustrate crawling of the MSC over the time period and by 120 minutes (F) the cell has stopped crawling, begun to spread and become phase dark. Around the periphery of the MSC are examples of fine phase-dark microvillous processes (or filopodia) (arrows) that extend during crawling of the MSC on the endothelial surface. Data are extracts of video S2 which is representative of 3 independent experiments. The bars represent 100 µm in A and 20 µm in B-F.

**Figure 2:**
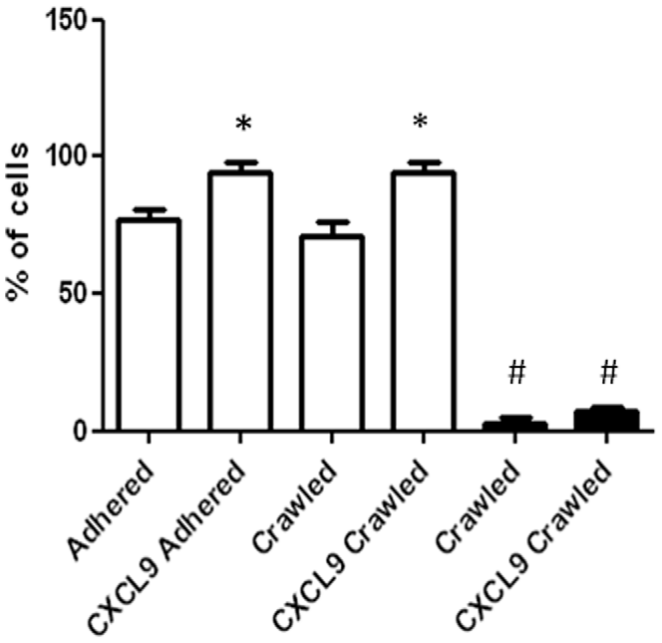
The percentage of mMSCs that adhered and crawled on MAECs. MAECs were TNFα-activated and incubated with or without CXCL9. mMSCs were then added, left for 10 minutes and media flowed over the MAECs for 2 hours and video recordings made as in Figure 1 (clear bars). mMSCs were also treated the same except that the flow was stopped during the 2 hour period (solid bars). Data are means (±standard errors) of 3 independent experiments (with passage number 7–9). *p<0.05 compared to the respective no chemokine control. #p<0.001 compared to same treatment under flow conditions.

Next mMSCs were added to MAECs which had been stimulated with TNFα alone (100ng/ml for 16 hours) or TNFα in the presence of CXCL9 (100 ng/ml for 30 min). The cells were left under flow-free conditions for 10min, and then the flow was started at 0.1 Pa (1 dyne/cm^2^) for 2 hours. MSCs appeared rounded and phase bright upon a background of endothelial cells (Figure 1). When the flow was started, 76% of the MSCs remained firmly adhered to the TNFα-treated endothelial cells whereas in the presence of CXCL9 and TNFα 94% remained firmly adhered, which was significantly more than in the absence of chemokine (p = 0.04) (Figure 2); the reminder of the MSCs detached and flowed away. Over the next 2 hours of flow, of those that had adhered 71% crawled on TNFα-treated endothelial cells whereas 94% crawled in the presence of CXCL9 and TNFα, amounting to a significant increase compared to the absence of chemokine (p = 0.02) (Figure 2). Crawling behaviour was characterised by the MSCs remaining phase bright and rounded in shape, undergoing lateral movement on the endothelial surface and extending fine microvillous processes (or filopodia) (Figure 1; videos S2 and S3). These filopodia were rapidly moving and associated with the MSC having a millipede-like movement. Quantitation revealed that mMSCs crawled 23±6 µm and 21±2 µm in the presence of TNFα and CXCL9/TNFα respectively prior to spreading (mean ± standard error of 3 independent experiments in each case) and there was no significant difference between these two values. In the presence of TNF, 37±7% of mMSCs crawled against the direction of flow and 60±8% crawled in the direction of flow. With CXCL9/TNF, 65±9% moved against the direction of flow and 36±9% in the direction of flow (data are means ± standard error of 3 independent experiments in each case). There was no significant difference between these values, in terms of TNF and CXCL9/TNF treatment and the direction of crawling.

**Figure 3:**
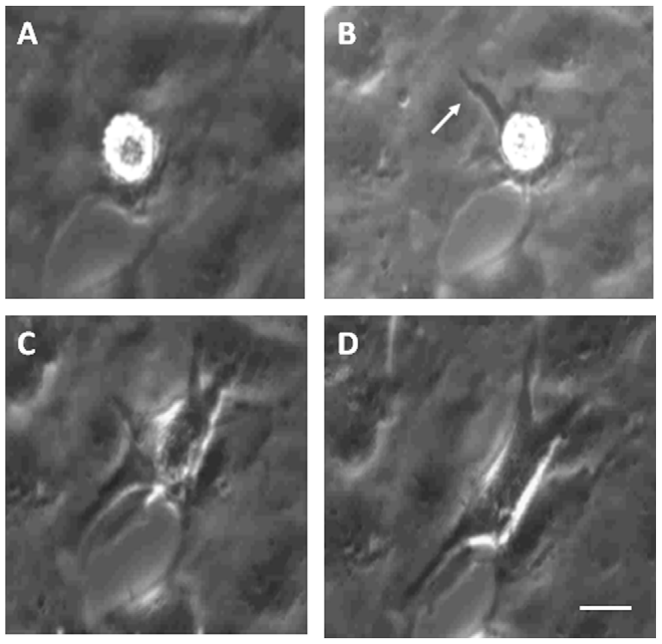
Still images from flow assay video to illustrate spreading of mMSCs in contact with MAECs. MAECs were TNFα-activated and incubated with CXCL9. mMSCs were then added, left for 10 minutes and media flowed for 2 hours and video recordings made. A-D show images the same MSC on TNFα-activated MAECs in the presence of exogenous CXCL9 (extracted from video S3). A, shows a rounded phase bright MSC after 30 minutes of flow. B, the cell is extending a pseudopod (arrow) after 60 minutes. C and D, the cell spreads becoming phase–dark extending pseudopods in several directions after 90 and 120 minutes respectively. Data are extracts of video S3 which is representative of 3 independent experiments. Bar represents 20 µm for each image.

**Figure 4:**
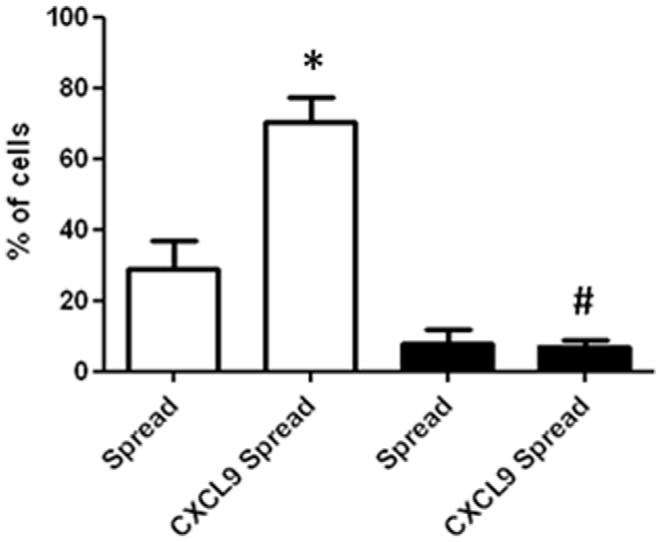
The percentage of mMSCs that spread in contact with MAECs. MAECs were TNFα-activated and incubated with or without CXCL9. mMSCs were then added, left for 10 minutes and media flowed over the MAECs for 2 hours and video recordings made as in Figure 3 (clear bars). mMSCs were also treated the same except that the flow was stopped during the 2 hour period (solid bars). Data are means (±standard errors) of 3 independent experiments with MSCs (with passage number 7-9). *p<0.05 compared to the respective no chemokine control. #p<0.001 compared to same treatment under flow conditions.

MSCs then spread out, becoming phase-dark and broad pseudopodia were extended in several directions often giving the MSC a stellate shape (Figure 3; video S3). 29% of the crawling MSCs spread with TNFα-treated MAECs, whereas 70% of crawling MSCs spread with TNFα and CXCL9-treated MAECs (Figure 4). This amounted to a significant increase in the percentage of spread MSCs in the presence of CXCL9 compared to its absence (p<0.001).

The influence of flow on the crawling and spreading of mMSCs on MAECs was then examined. mMSCs were added to the endothelial cells and left under flow-free conditions for 10 min. The flow was started briefly for 15 seconds to remove non-adherent cells. It was then stopped and the adhered cells videoed for 2 hours to image crawling and spreading behaviour. In terms of crawling, only 3% of adhered mMSCs crawled on TNFα-activated MAECs in the absence of flow, increasing to 8% with the addition of exogenous CXCL9 (Figure 2). These values were significantly less than in presence of flow (p<0.001 in each case), amounting to 24- and 12-fold reductions respectively. In terms of spreading, 8% of MSCs spread with TNFα-treated MAECs in the absence of flow which was not significantly different (p = 0.08) than in the presence of flow (28%) (Figure 4). With the addition of CXCL9, 7% of mMSCs spread in the absence of flow which was significantly less than in the presence of flow (70%), amounting to a 10-fold reduction (p<0.001) (Figure 4).

### Murine Mesenchymal Stem Cell Migration across an Endothelial Layer is Increased in Response to Chemokines

In order to examine if the mMSCs transmigrated across endothelial cells, MAECs were grown on the filters of transwells. Chemotaxis assays were set up as described with 10ng/ml CCL20, CCL25, CXCL9 or CXCL16, or medium alone, in the bottom well after treatment of MAECs with murine TNFα. Three wells were left untreated as a control. To work out the number of cells per well which had migrated across the endothelium in the transwell system, a standard curve of fluorescence against cell number was set up, using some of the same population of mMSCs that had been labelled with calcein for the assay. Each set of conditions was set up in triplicate, and three fluorimeter readings were taken for each well to calculate the number of cells which had migrated through.

**Figure 5:**
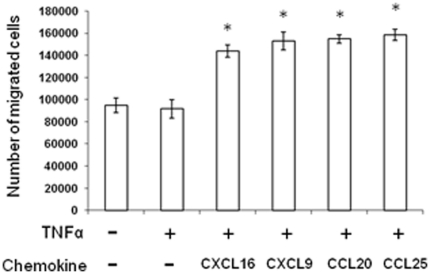
Transendothelial migration of mMSCs in response to chemokines. Graph to show number of fluorescently labelled MSCs that migrated to the bottom well through MAEC layers grown on the filters of transwell inserts. MAECs were treated with or without TNFα and in the presence or absence of CXCL16, CXCL9, CCL20 and CCL25 (each at 10 ng/ml). Values represent means ± standard errors of 3 independent experiments and each experiment was performed in triplicate. *p<0.0001 compared to control in the presence of TNF and absence of chemokine and p = 0.0004 compared to control in the absence of TNF and chemokine.

A significantly higher number of mMSCs migrated across the MAEC layer stimulated with TNFα, into the bottom well, in response to CXCL16 (mean of 143807 cells per well), CXCL9 (mean of 153083 cells per well), CCL20 (mean of 154894 cells per well) and CCL25 (mean of 158640 cells per well), compared to TNFα stimulated MAECs with no chemokine (mean of 91785 cells per well) or MAECs with no TNFα stimulation and no chemokine (mean of 94868 cells per well) (Figure 5, *P = *<0.001 for all four chemokines with TNFα, versus no chemokine with TNFα, or media alone).

### Analysis of Migrated mMSCs

**Figure 6:**
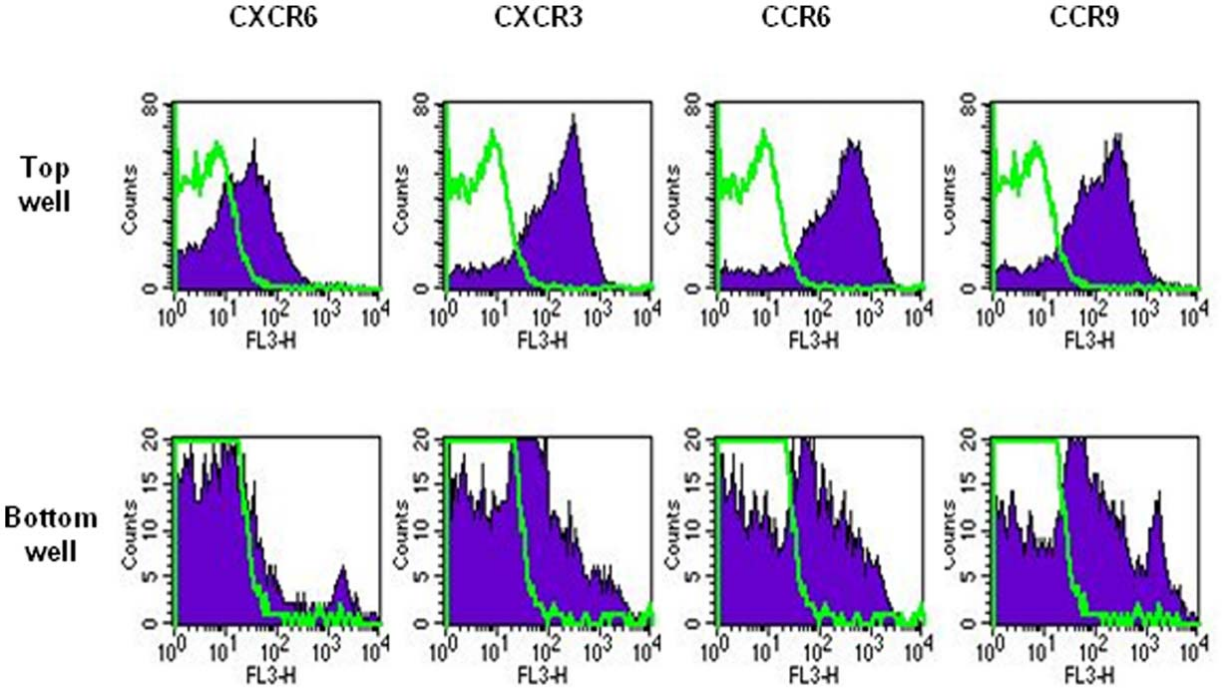
Migrated mMSCs show a lower level of chemokine receptor expression. Histogram plots to show chemokine receptor expression on mMSCs in the top and bottom wells of transwells after transendothelial chemotaxis. Percent positive mMSCs and mean fluorescence intensity (MFI) are shown for each plot. Line graphs are Ig controls and filled histograms show results with chemokine receptor antibodies. Data show a representative experiment.

The migrated and non-migrated mMSCs were also analysed by flow cytometry for expression of the chemokine receptor corresponding to the chemokine that was used in the assay. This was in order to study if gene expression was altered as a result of transmigration. The chemokine receptors CXCR3, CXCR6, CCR6 and CCR9 were found to be down-regulated on the migrated cells compared to those left in the top well, both in terms of their mean fluorescence intensity (MFI) and percent positive cells (Figure 6 and Table 1). Murine MSCs which had migrated in response to CXCL16 were 20% positive for CXCR6 compared to 55% in the top well, and had an MFI of 10 compared to 20. Murine MSCs which had migrated in response to CXCL9 were 46% positive for CXCR3 compared to 89% in the top well, and had an MFI of 25 compared to 117. Murine MSCs which had migrated in response to CCL20 were 53% positive for CCR6 compared to 91% in the top well, and had an MFI of 33 compared to 198. Murine MSCs which had migrated in response to CCL25 were 51% positive for CCR9 compared to 88% in the top well, and had an MFI of 46 compared to 97.

**Table 1:** Down-regulation of chemokine receptors on mMSCs in transwells after transendothelial migration.

	CXCR6	CXCR3	CCR6	CCR9
Top well (% positive cells)	55%	89%	91%	88%
Bottom well (% positive cells)	20%	46%	53%	51%
Top well (MFI)^a^	20	117	198	97
Bottom well (MFI) a	10	25	33	46

Data are obtained from Figure 6;

a =  mean fluoresence intensity.

## Discussion

The current study showed that mMSCs interact with MAECS showing firm adhesion, and crawling and spreading behaviour, which were all enhanced by CXCL9. These interactions occurred when the mMSCs were applied to the endothelial cells, left under shear–free conditions for several minutes and then the flow started. However, under conditions of constant shear flow we observed no capture or rolling of mMSCs on the surface of MAECs or bEnd.3 endothelial cells stimulated with TNFα, chemokines or both, and at two different wall shear stresses (0.1 and 0.05 Pa (1 and 0.5 dynes/cm^2^)). This is in contrast to leukocytes such as neutrophils that show rolling behaviour in the same in vitro flow assay system [35]. The lack of rolling of MSCs is probably related to the lack of expression of L-selectin and the P- and E-selectin counterligands glycoprotein ligand-1 (PSGL-1) and sialyl Lewis X carbohydrates, reflecting the finding that MSCs are unable to bind functionally to constructs of P- and E-selectin [13], [25], [36], [37]. Such counterligands are expressed on all leukocytes, and mediate the rolling phase of the adhesion cascade by interacting with selectins on endothelial cells [14]. Others have also shown a lack of rolling behaviour of MSCs on stimulated and unstimulated human umbilical vein endothelial cells (HUVECs) in vitro under flow conditions [37]. One study has shown rolling MSCs on HUVECs in vitro, however rolling velocities were high, 100–500 µm/s at shear stress of 0.1–1.0 dynes/cm^2^ [13], [25]. For comparison, leukocytes typically roll at around 5 µm/s at shear stresses of up to 4 dynes/cm^2^ [38].

There are two potential mechanisms for how MSCs may decelerate within the vasculature during the extravasation process [13]. Firstly their large size may reduce their velocity due to physical interactions with narrower capillaries leading to arrest and passive entrapment. It has been noted that when MSCs are injected intravenously they can become trapped in organs such as the lungs, even under normal conditions in the absence of tissue damage or inflammation, and this has been attributed to their large size [39]. Secondly, similar to leukocytes, MSCs may actively tether and roll on the activated vasculature leading to arrest and firm adhesion. Our results are in line with the first mechanism suggesting that it is not an obligatory prerequisite to have leukocyte-like rolling prior to arrest and firm adhesion. As long as the MSCs are slowed or stationary for a period of time it appears to be sufficient to enable chemokine presentation and firm adhesion, followed by crawling and spreading.

There are several clinical situations when blood flow is stopped for a period of time for the treatment of atherosclerosis. For example this occurs during coronary artery bypass surgery and angioplasty when a balloon catheter is inflated in the artery. In stem cell trials for the treatment of atherosclerosis it is worth noting that a period when the blood flow is stopped and then restarted may not be detrimental to the recruitment of these cells. In addition, deceleration of MSCs in the small vessels of the artery wall (eg in the vasa vasorum) in arteriosclerosis may be sufficient for recruitment of these cells.

In the current study stopping the flow for several minutes prior to starting it resulted in shear-resistant arrest of the MSCs on the endothelial cell surface. This resulted in virtually all of the MSCs (94%) remaining attached in the presence of chemokine. It is probable that integrins such as VLA-4 is involved in the firm attachment since this has been shown to be expressed by MSCs and to mediate adhesion to V-CAM on the endothelial surface [25], [26], [28], although this may depend on the tissue source of the MSCs [36]. The finding that CXCL9 enhances the firm adhesion of mMSCs to MAECs suggests that this chemokine is presented to the MSC resulting in stimulation of integrin activation and increased affinity of this adhesion molecule for its endothelial ligand. In this respect integrin activation is a classic role of chemokines, enhancing the firm adhesion of leukocytes to the endothelium [14].

In the case of leukocytes, they do not necessarily transmigrate at the point of initial arrest but locomote laterally, or crawl, to preferred sites of transendothelial migration [40]. Lymphocytes in vitro extend filopodia and undergo millipede-like crawling on endothelial cells which is enhanced by exogenous chemokine [41]. In the present study MSCs also extended fine microvilli or filopodia and underwent millipede-like crawling, furthermore exogenous CXCL9 increased the percentage of MSCs that crawled. They were able to crawl against or with the direction of flow, which has also been reported for neutrophils [42], and the distance crawled was similar to that of shown for leukocytes in vitro, around 20 µm [40]. Following crawling, MSCs spread, changing from a spherical morphology to a flattened often stellate shape. In this respect spreading is also a feature of migrating leukocytes [14], [43]. Exogenous CXCL9 particularly increased the numbers of MSCs that spread in comparison with the other parameters measured. Spreading was characterised by the extension of pseudopodia in multiple directions. These structures resemble the plasmic podia which have also been described on human MSCs interacting with cultured endothelial cells under static conditions [27].

In the present study endothelial cells in the flow assay were pre-treated with TNF which may up-regulate expression of their adhesion molecules and chemokines. Addition of exogenous CXCL9 increased MSC adherence, crawling and spreading on endothelial cells, in comparison to its absence, showing a significant effect of this chemokine. However, it cannot be ruled out that endothelial-expressed chemokines may be having an effect as well, so displacement of such chemokines by heparin for example may have given a better indication of the role of CXCL9. Interestingly, in the case of transwell migration experiments TNF treatment alone of the same endothelial cells does not appear to be having an effect on adhesion and transmigration since the number of migrated MSCs is the same in the presence or absence of TNF.

Arrest of leukocytes on endothelial cells is enhanced by flow-derived shear forces [44], [45]. It is indicated that this is mediated by shear enhancing the activation of integrins which occurs in association with the action of chemokines. In the case of MSCs, these cells were particularly sensitive to the effect of shear, when the flow was stopped during the phases of crawling and spreading there was a marked reduction in the numbers of MSCs showing such behaviour. In the absence of flow the percentage of crawling MSCs was negligible (3%) increasing to 71% in the presence of flow, and this increased to 94% in the presence of flow and chemokine. This indicates that flow is a major contributing factor in addition to that of chemokine. Similarly spreading was markedly increased by shear forces with the percentage of cells showing this behaviour increasing from 7% to 70% in the presence of chemokine. Therefore the influence of flow needs to be taken in consideration in designing therapeutic strategies when MSCs are administered in the circulation.

There have been many in vivo studies in disease models and humans showing that MSCs administered into the circulation can engraft into the affected tissues, although the efficiency may be quite low [1], [13]. For example this occurs following myocardial infarction where MSC recruitment into the heart can result in clinical benefit [1], [46]. However, very few studies have examined the transmigration of MSCs. Steingen et al [27] reported that MSCs can transmigrate across non-activated endothelial monolayers via VCAM/VLA-4, but rather than undergoing complete diapedesis, as observed for leukocytes, MSCs tended to integrate in to the endothelial cell layer, possibly as embedded pericytes. The current study shows that MSCs can fully transmigrate across the endothelial cell layer, into the bottom well of transwells, and that this is significantly enhanced by the presence of chemokines CXCL9, CXCL16, CCL20 and CCL25. These chemokines were chosen since they are the ligands for the receptors CXCR3, CXCR6, CCR6 and CCR9 which were shown to be particularly expressed on the mMSCs used in the present study [17]. The chemokines showed specificity in their effects since in their absence (no chemokine control), significantly less MSCs transmigrated. In addition, TNF alone did not enhance MSC migration compared to control further indicating the specificity of the chemokine response. In our previous study [17] using standard Boyden-style chemotaxis assays in the absence of endothelial cells CXCL9, CXCL16, CCL20 and CCL25 (at 10 and 100 ng/ml) elicited a migratory response using the same mMSCs, whereas CXCL12 did not. This further showed specificity of the migratory responses of mMSCs to chemokines.

The chemotactic responses of mMSCs in Boyden-style chambers are comparatively sensitive, peaking between 10 and 100 ng/ml and showing lack of response above and below these concentrations [17]. In the case of CCL25, higher concentrations have been required to stimulate chemotaxis (eg 1500 ng/ml) using other cells types such as human Jurkat cells [47]. In the current transendothelial migration study 10 ng/ml of chemokines was used leading to significant effects on mMSC migration. This further suggests that mMSCs are relatively sensitive to migratory stimulation by chemokines although the effects of having endothelial cells may also enhance migration compared to chemotactic filters.

In addition, following the adhesion and transmigration of MSCs across endothelial cells gene expression is altered and chemokine receptors are down-regulated. Ligand-induced down-regulation is a well-documented feature of leukocyte chemokine receptors occurring by receptor internalization and endocytosis [48]. The down-regulation on MSCs suggests that once these cells have migrated across the endothelium and into the tissue, chemokine receptor expression may be reduced or a different spectrum of chemokine receptors induced in the tissue environment, as indicated for lymphocytes [49], influencing further positioning within the tissue.

In conclusion the model derived from the current study has aspects of both passive and active homing previously proposed for MSCs [13]. Passive reduction of velocity or stasis of MSCs may occur when they pass though narrow capillaries, this enables chemokine presentation, integrin activation, arrest and firm adhesion, crawling and spreading. The MSCs then follow a directional cue given by immobilised chemokines to migrate into the extravascular tissue. Since CXCR3 and CXCR6 are reported to be important for T lymphocyte and monocyte recruitment to atherosclerotic plaques [50], [51], MSCs may be able use these receptors to enter the lesion. Furthermore manipulating the expression of these receptors on MSCs may enable increased recruitment as a therapeutic benefit in the disease.

## Supporting Information

Video S1 **mMSCs flowing over TNFα stimulated MAECs, with exogenous CXCL16 added, after 4 minutes of continuous flow.** There is lack of interaction of mMSCs with the endothelial cell layer, with MSCs appearing as blurred “streaks.” The video is representative of 3 independent experiments. Magnification ×200.
(MPEG)

Video S2 **mMSCs interacting with TNFα stimulated MAECs.** MSCs were incubated with the endothelial cells for 10 minutes in the absence of flow. The flow was then started and continued for 2 hours. The video shows adhered phase-bright MSCs under flow that undergo crawling, extending numerous fine microvilli that rapidly change shape on the endothelial surface. Some of the MSCs then spread becoming phase-dark. The video is representative of 3 independent experiments. Bar  = 200 µm.
(MPEG)

Video S3 **mMSCs interacting with TNFα stimulated MAECs with exogenous CXCL16 added.** MSCs were incubated with the endothelial cells for 10 minutes in the absence of flow. The flow was then started and continued for 2 hours. The video shows adhered MSCs under flow that undergo crawling when phase-bright and then spreading becoming phase-dark. The video is representative of 3 independent experiments. Bar  = 200 µm.
(MPEG)
